# Assessing the Impact of Multigene Engineering on the Proteome: Omega‐3 Camelina as a Case Study

**DOI:** 10.1111/pbi.70712

**Published:** 2026-07-13

**Authors:** Hongtao Zhang, Johnathan A. Napier, Frederica L. Theodoulou

**Affiliations:** ^1^ Rothamsted Research Harpenden UK

**Keywords:** data independent acquisition, isobaric labelling, lipid metabolic engineering, oil yield penalty, proteomics, proteostasis

*Camelina sativa* (Camelina) has emerged as a valuable platform for lipid metabolic engineering owing to its short life cycle, favourable seed oil profile and amenability to genetic transformation (Haslam et al. 2025). Previous studies have demonstrated the successful installation of non‐native pathways for the biosynthesis of the omega‐3 very‐long‐chain polyunsaturated fatty acids, eicosapentaenoic acid (EPA) and docosahexaenoic acid (DHA) in seeds (Han et al. 2020, 2022; Figure S1). However, the broader consequences of introducing such a complex, multi‐step pathway on endogenous processes remain largely uncharacterised.

In common with other lipid metabolic engineering studies, EPA and DHA production is associated with a reduction in seed oil content‐ the so‐called oil yield penalty, which is poorly understood (Bates et al. 2014; Han et al. 2020). Moreover, studies in other engineered crops suggest that manipulating even a single gene can lead to substantial proteome reprogramming (Alvarez et al. 2015), highlighting the potential for both targeted and unintended effects on the proteome. Understanding these impacts is essential for evaluating the stability, performance and commercial viability of engineered crops. Quantifying transgene‐encoded proteins is also important for understanding pathway performance.

Tandem mass tag (TMT)‐based multiplexing and data independent acquisition (DIA)‐based label‐free quantification (LFQ) offer different advantages for profiling both endogenous and heterologous proteins: TMT workflows provide precise cross sample relative quantification, while DIA plus intensity‐based absolute quantification (iBAQ) provides broader coverage and approximate absolute abundance estimates, allowing robust cross validation of differential abundance and pathway enrichment as well as stoichiometric insight. Here, we apply these complementary strategies to characterise the seed proteomes of elite EPA‐ and DHA‐producing Camelina lines which differ in their oil yield penalty. DHA1 and EPA8 typically exhibit an oil yield penalty of 8 and 5 percentage points, respectively (Han et al. 2020, 2022; Figure S1). Our goals were to assess global proteomic changes associated with multigene engineering, to identify proteomic signatures associated with the differing oil yield penalties observed in EPA and DHA lines, and to quantify the relative abundance of transgene‐encoded enzymes.

We profiled proteomes of wild type and engineered seeds (WT, DHA1 and EPA8 lines; Figure S1) at the mid‐seed filling stage, 25 days after pollination, when the oil yield penalty is already established (Han et al. 2020). Gel electrophoresis revealed distinct protein profiles, with markedly reduced seed storage protein abundance in the engineered lines, particularly DHA1 (Figure 1a). TMTpro16‐plex‐based analysis (Figure S2) identified 8239 Camelina proteins and eight transgene‐encoded proteins, with relative quantitation for 4955 proteins (Data S1; Figure S3). Principal component analysis revealed clear separation of WT from both transgenic lines (Figure 1b). In DHA1 seeds, 317 proteins were increased and 424 decreased in abundance relative to WT (FC > 2; *p*_adj_ < 0.05); markedly fewer proteins were differentially abundant in EPA8 (Figure 1c). Proteins with increased abundance in DHA1 were enriched for KEGG pathways associated with fatty acid degradation (β‐oxidation and the glyoxylate cycle), whereas proteins involved in fatty acid biosynthesis and photosynthesis were decreased in abundance (Figure 1d,e; Figure S4). Simultaneous biosynthesis and degradation of fatty acids occurs through all phases of oil filling in Camelina, particularly in lines engineered for high seed oil content (Koley et al. 2025); interestingly, in our study, the more pronounced changes in DHA1 lipid metabolic enzymes relative to EPA8 align with its greater oil yield penalty. However, it should be acknowledged that this study does not distinguish between pathway‐specific and event‐specific effects. Proteasome associated proteins were also upregulated in DHA1 (Figure S5), perhaps indicating enhanced protein turnover to balance the dysregulated proteome. In wheat, 25% of newly synthesised storage proteins are turned over during grain development (Cao et al. 2022), consistent with accumulation of transgene encoded proteins associated with reduced seed storage protein abundance in Camelina (Figure 1).

**FIGURE 1:**
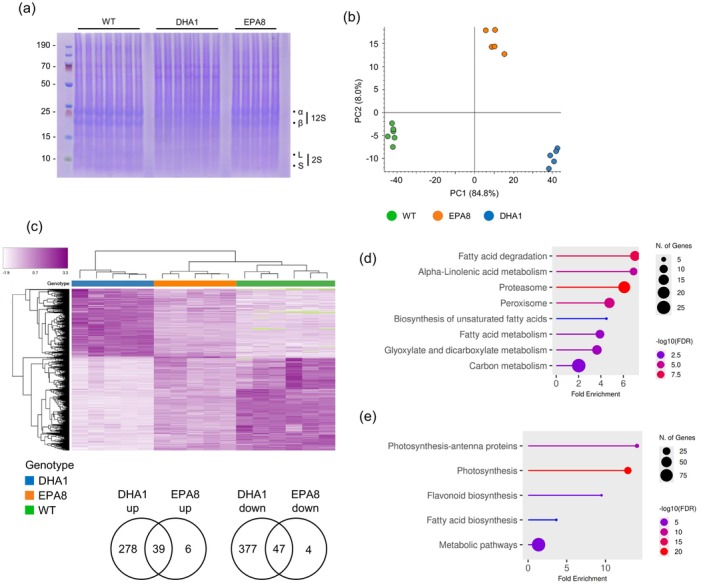
Impact of transgene‐encoded EPA and DHA biosynthetic pathways on the Camelina seed proteome. (a) Protein profile of seeds collected 25 days after pollination; positions of 2S and 12S seed storage proteins are indicated to the right. (b) Principal component analysis of normalised protein abundances. (c) Heatmap showing normalised scaled abundances of differentially abundant proteins (FC > 2; *p*_adj_ < 0.05) and Venn diagrams of differentially abundant proteins in DHA1 and EPA8 seeds. (d, e) Enrichment of pathways with increased (d) and decreased abundance (e) in the DHA1 line.

Using a DIA workflow (Figure S6), we identified a total of 7835 protein groups, including 9 transgene‐encoded proteins (Figures S7a and S8, Data S2). The three genotypes were clearly separated by multidimensional scaling, with fewer proteins per sample identified in WT seeds, reflecting the relatively higher levels of seed storage proteins (Figure S7b,c). Of the 5860 proteins quantified in both DHA1 and WT by LFQ, 755 proteins exhibited 2‐fold significant changes, compared to only 75 of the 5792 proteins quantified in EPA8 and WT (FC > 2; *p*_adj_ < 0.01; Figure S7d; Data S2). DIA and TMT workflows identified distinct but overlapping sets of proteins, with 4616 proteins identified in both datasets (Figure S7e). KEGG pathway enrichment in DHA1 seeds was very similar to that for the TMT experiment (Figure S7f,g), with DIA additionally identifying proteins enriched in alkaloid and tyrosine metabolic pathways. The strong correlation of protein fold changes between the two different quantification methods (Figure S7e,h) underlines the robustness of the findings.

To quantify transgene‐encoded proteins and estimate their stoichiometry with lipid biosynthetic pathway components, we analysed the label‐free DIA data using iBAQ. The dynamic range of the observable Camelina seed proteome spanned 6 orders of magnitude (Figure S9a). DIA identified 86 endogenous lipid biosynthetic proteins with relatively high abundances (Figure S9b). The soluble, stable marker protein DsRed was the most abundant transgene‐encoded protein with high peptide coverage, but the ER resident lipid biosynthetic enzymes exhibited lower coverage and PerfΔ15 was not detected in multiple mass spectrometry runs (Figures S8 and S9c,d). Although driven by strong promoters, the abundance of heterologous elongases and desaturases varied over a 200‐fold range (Figure S9; Data S2), comparable to endogenous lipid biosynthetic enzymes such as acetyl CoA carboxylase complex subunits which exhibit sub‐optimal stoichiometry in vivo (Wilson and Thelen 2018). These findings highlight challenges in detecting and quantifying heterologous ER‐resident enzymes and achieving balanced pathway expression.

In conclusion, our study underscores the value of quantitative proteomics for understanding proteome remodelling, diagnosing potential pathway bottlenecks (e.g., linking the oil yield penalty to enhanced β‐oxidation), and guiding future construct design, including promoter choice, expression strength and stoichiometric balancing. The datasets also offer a quantitative framework for regulatory assessment of intended and unintended effects in stacked traits, providing clear applied value for supporting the deployment of engineered Camelina lines.

## Author Contributions

Conception: F.L.T. and H.Z.; experimentation: H.Z.; data analysis: H.Z. and F.L.T.; materials: J.A.N.; writing: H.Z. and F.L.T; manuscript review: J.A.N.

## Funding

This work was supported by Biotechnology and Biological Sciences Research Council (BB/X010988/1, IGP22‐035).

## Conflicts of Interest

J.A.N. has acted as a consultant for Yield10 Bioscience and BASF and is an inventor on patent families associated with the production of EPA and DHA in transgenic plants. F.L.T. is a non‐executive director of SugaROx Ltd.

## Supporting information


**Data S1:** TMTpro 16‐plex dataset.

**Data S2:** DIA dataset.

**Methods S1.** Materials and methods.

**Figure S1:** Details of transgenic lines.
**Figure S2:** Tandem mass tag (TMT) label‐based quantitative proteomics workflow.
**Figure S3:** Transgene encoded proteins identified using TMT label‐based proteomics.
**Figure S4:** Differential abundance of lipid metabolic enzymes in Camelina seed engineered to produce EPA and DHA.
**Figure S5:** Differential abundance of ubiquitin proteasome‐related proteins in Camelina seed engineered to produce EPA and DHA.
**Figure S6:** DIA label‐free quantitative proteomics workflow.
**Figure S7:** Camelina seed proteins identified and quantified using MaxLFQ label‐free quantification.
**Figure S8:** Transgene encoded proteins identified using label‐free proteomics.
**Figure S9:** Normalised intensity‐based absolute quantification (iBAQ) of Camelina seed proteins.

## Data Availability

Proteomics data have been deposited to the ProteomeXchange Consortium via the PRIDE partner repository with the dataset identifiers PXD074097 (TMT) and PXD074106 (DIA).
