# Supplementary material for: Assessing the Impact of Multigene Engineering on the Proteome: Omega‐3 Camelina as a Case Study
Source: Plant Biotechnol J. 2026 Jul 13;24(10):5480–2. doi: 10.1111/pbi.70712 (PMC13399257; doi:10.1111/pbi.70712)
Supplement: Supplementary file 1 [file PBI-24-5480-s002.docx]

**Supplemental Data**

**Materials and Methods**

*Plant material*

Transgenic *Camelina sativa* lines expressing DHA2015.1 (DHA1; Han et al., 2020) and EPA2015.8 (EPA8; Han et al., 2022), together with wild type (WT) controls, were grown under controlled environment (CE) conditions: 16 h photoperiod, 23 °C, 65 % relative humidity, and 400 µmol m⁻² s⁻¹ LED illumination during the day and 18 °C and 75 % relative humidity at night. Developing seeds were collected at 25 days after pollination (DAP), flash frozen in liquid nitrogen, and stored at −80 °C. 25 DAP was selected because lipid accumulation has plateaued at this developmental time point (Pollard et al., 2015), the oil yield penalty has been established (Han et al., 2020), seed storage proteins are abundant (Nguyen et al., 2013; Russo and Reggiani, 2015) and the transgene-encoded proteins are driven by seed storage protein promoters which are typically active at this stage of seed development.

*Protein and peptide sample preparation*

Samples were randomised for protein extraction. Seeds were ground using a GenoGrinder (17,500 rpm, 2 min) with cooling, and 30–50 mg of powder was used per extraction. Samples were resuspended in 400 µl extraction buffer containing 8 M urea, 50 mM TEAB, and 1 X Complete Mini Protease Inhibitor Cocktail (Roche), 1 X PhosStop Phosphatase Inhibitor Cocktail (Roche), incubated on ice for 30 min, and centrifuged (13,000 rpm, 15 min, 4 °C). Proteins (400 µg) were reduced with 10 mM TCEP for1 h at 55 °C, alkylated with 17 mM iodoacetamide for 30 min in the dark. The protein was precipitated by methanol/chloroform, washed, and resuspended in 100 mM TEAB as previously described in Zhang et al. (2018). Digestion was performed with sequencing grade trypsin (1:100 trypsin : Camelina protein ratio) for 4 h, followed by additional trypsin (final 1:50) overnight at 37 °C. Proteins and peptides were visualised on 4-12 % Bis-Tris Midi Protein Gels (WG1403box, Thermo Scientific) stained with Coomassie Brilliant Blue. Peptide concentration was determined using a Pierce Quantitative Colorimetric Peptide Assay kit (23275, Thermo Scientific).

*TMTpro™ Labelling and Fractionation*

Peptides (10 μg each sample) were labelled with 50μg TMTpro™ 16 plex reagents (A58334; WT: 126–129N; DHA1: 129C–131; EPA8: 132–134), according to manufacturer’s instructions, pooled and dried. 60 μg peptides were fractionated into eight fractions using a Pierce High pH Reversed-Phase Peptide Fractionation Kit (84868). Peptide concentration was determined using a Pierce Quantitative Colorimetric Peptide Assay kit.

*LC–MS/MS Analysis of TMTpro™ labelled samples*

About 500 ng of each fraction was analysed on an Orbitrap Eclipse™ Tribrid™ mass spectrometer coupled to a Vanquish Neo UHPLC in nano capillary flow. Peptides were separated on a 25 cm × 75 µm 1.7 µm C18 column (IonOpticks) using a 180 min gradient at 0.4 µL/min (3–45 % B; mobile phase B: 80 % acetonitrile + 0.1 % formic acid). The eluted peptides were sprayed into the mass spectrometer by means of an Nanospray Flex™ ion source (Thermo Fisher Scientific Inc.). The instrument was operated in positive ion mode with a spray voltage of 1.9 kV and an ion transfer tube temperature of 275 °C. All m/z values of eluting peptide ions were measured in an Orbitrap mass analyser, set at a resolution of 120,000 and were scanned between m/z 400-1600 Da. Quadrupole isolation was enabled, with an automatic gain control (AGC) target of 4×10⁵; was used to filter low‑abundance precursors. Data‑dependent MS2 scans were acquired in the ion trap using collision‑induced dissociation (CID) fragmentation. Precursors were isolated with a 0.7 m/z window in the quadrupole and fragmented by CID at 30 % normalized collision energy, with an activation time of 10 ms and activation Q of 0.25. MS2 spectra were acquired in the ion trap at turbo scan rate, with an AGC target of 1×10⁵; and a maximum injection time of 35 ms. Spectra were recorded in centroid mode. TMTpro™ tag‑loss exclusion was enabled, and precursor ion exclusion was applied with ±25 ppm mass width.

MS3 scans were acquired in the Orbitrap using higher-energy collisional dissociation (HCD) fragmentation with synchronous precursor selection (SPS). Ten SPS precursors were selected. MS2 isolation used a 2 m/z window and MS3 isolation used a 0.7 m/z window. HCD fragmentation was performed at a normalized collision energy of 65 %. MS3 scans were collected at 50,000 resolutions over m/z 100–500 with an AGC target of 1×10⁵ (200 % normalized), a maximum injection time of 200 ms, one microscan, and centroid data output. This was performed in cycles of 10 MS3 events before the instrument reverted to scanning the m/z ratios of the intact peptide ions and the cycle continued. When Real‑time search (RTS) was on, searching was enabled using a tryptic search against Cs_v3_epa8_dha database 98271 hits downloaded from https://cruciferseq.ca/Csativa_download and 10 proteins encoded by transgenes in DHA1 and EPA8. Static modifications included carbamidomethylation on cysteine (+57.0215 Da) and TMTpro™16plex on lysine and peptide N‑termini (+304.2071 Da). Oxidation of methionine (+15.9949 Da) was included as a variable modification. One missed cleavage and up to two variable modifications per peptide were allowed. FDR filtering was disabled. The maximum search time was 35 ms. SPS‑MS3 triggering was enabled with scoring thresholds of Xcorr ≥1.4, dCn ≥0.1, precursor mass error ≤10 ppm, and charge state 2.

*LC–MS/MS analysis of label free samples*

500 ng of each peptide was analysed on an Orbitrap Eclipse™ Tribrid™ mass spectrometer coupled to a Vanquish Neo UHPLC system using a 25 cm × 75 µm 1.7 µm C18 column (IonOpticks) operated at a flow rate of 0.4 µL/min. Samples were loaded at 1500 bar with the autosampler maintained at 7 °C. The 65‑minute LC gradient was as follows: 0–30 min at 3 % B, 30–40 min from 3 % to 17% B, 40–50 min from 17 % to 25 % B, 50–57 min from 25 % to 30 % B, and 57–65 min from 30 % to 35 % B, followed by a column wash and re‑equilibration.

The eluted peptides were sprayed into the mass spectrometer by means of an Nanospray Flex™ ion source (Thermo Fisher Scientific Inc.). The instrument was operated in positive ion mode with a spray voltage of 1.9 kV and an ion transfer tube temperature of 275 °C. The system operated in LC infusion mode with an expected chromatographic peak width of 10 seconds, advanced peak determination enabled, and a default precursor charge state of 2. Lock mass correction was disabled.

Data‑independent acquisition (DIA) was performed throughout the 65‑min run. The DIA master scan covered a precursor mass range of m/z 400–900 using quadrupole isolation. The method used automatically defined DIA windows with a nominal width of 12 m/z and a 1 m/z overlap, resulting in 42 sequential scan events. Window placement optimization was enabled. Fragmentation was performed using HCD with a normalized collision energy of 30 %. DIA MS2 spectra were acquired in the Orbitrap at a resolution of 15,000 over an m/z range of 145–1450. The AGC target was set to 4×10⁵ (800 % normalized) with automatic maximum injection time, one microscan, centroid data output, and positive polarity. Source fragmentation remained disabled.

*Data Processing and Statistical Analysis*

Raw TMT MS data were processed in Proteome Discoverer 3.0.0.757 using a workflow optimized for TMTpro™ 16plex SPS‑MS3 quantification with real‑time search. Spectra were filtered to retain MS2 scans with valid precursor assignments and charge states 2-6. Database searching was performed in parallel with Sequest HT, Comet, and Mascot2.8 against the Cs_v2_epa8_dha_combined database 92469 hits downloaded from <https://cruciferseq.ca/Csativa_download> and 10 proteins encoded by transgene in DHA1 and EPA8, supplemented with 298 common contaminants PD_Contaminants_2015_5 provided by the manufacturer. Search was set as Trypsin enzyme specificity with a maximum of one missed cleavage, precursor tolerances was set as 10 ppm, fragment tolerances of 0.6 Da. Carbamidomethylation (+57.021 Da) of cysteine and TMTpro™ isobaric labelling (+304.207) of lysine and N-termini were set as static modifications while the methionine oxidation (+15.996) were considered dynamic.

Sequest HT PSMs were rescored using INFERYS, and all search engines were evaluated using Percolator with a concatenated target–decoy strategy. Peptide‑spectrum matches were filtered to a 1 % FDR at the PSM level. Reporter ion intensities were extracted from FTMS MS3 HCD scans using a 20‑ppm integration tolerance and the most confident centroid method. Quantification and statistical analyses were performed in PD3.0. Peptide‑level quantification used unique + razor peptides, with protein‑group-aware peptide uniqueness enabled. Shared peptide quantification was retained, and channels with missing values were not excluded. Reporter ion abundances were determined automatically with correction factors applied according the TMTpro™ product sheet 16plex_YK388744, using thresholds of ≥10 average S/N, ≥65 % SPS mass matches, co‑isolation <50 %, and a normalized CHIMERYS coefficient ≥0.8. Quantitative values were normalized using the Total Peptide Amount method and scaled across all channels. All peptides were included for normalization and protein roll‑up, while modified peptides were excluded from pairwise ratio calculations. Protein abundances were calculated using a protein‑abundance–based roll‑up, with a maximum allowed fold change of 1000. No imputation was applied. Statistical significance was assessed using ANOVA at the individual‑protein level.

RAW files of DIA data search and legacy (direct) quantification were performed using DIA-NN (version 2.2.0; Demichev et al., 2022). The same Cs_V2_EPA_DHA database was used for library-free search/library generation. For RT prediction and extraction mass accuracy, we used the default parameter 0.0, which means DIA-NN performed automatic mass and RT correction. The FDR was set to 1% at the peptide precursor level. The variable modifications allowed were as follows: N term acetylation and Oxidation (M). The fixed modifications allowed were carbamidomethylation of cysteine. Search was set as Trypsin enzyme specificity with a maximum of one missed cleavage. The report.parquet file was processed using DIAgui v1.4.8 (Gerault et al., 2024). Intensities quantification was generated with “Precursor.Normalized' using MaxLFQ from iq package, with q-value <0.01 set for protein and protein group and quantity quality >0.8 and PEP <0.05. The iBAQ score was calculated based on the raw quantities from the columns 'Precursor.Quantity'. The normalized iBAQ was calculated as in Zhong et al. (2025).

*GO terms enrichment*

Gene Ontology (GO) terms and KEGG pathway enrichment were performed using Pathview and ShinyGO 0.85.1 (Ge et al., 2020), with the Arabidopsis homologues of all identified Camelina proteins as background.

*Use of AI*

Copilot was used to provide suggestions for shortening the main text. AI-generated text was carefully reviewed and edited by the corresponding author prior to inclusion in the manuscript.

*Data availability*

The mass spectrometry proteomics data have been deposited to the ProteomeXchange Consortium via the PRIDE partner repository (Perez-Riverol et al., 2022) with the dataset identifiers PXD074097 (TMT) and PXD074106 (DIA).

**References**

Demichev V, Messner CB, Vernardis SI, Lilley KS, Ralser M. (2020) DIA-NN: neural networks and interference correction enable deep proteome coverage in high throughput. Nat Methods. **17**, 41-44.

Ge SX, Jung D, Yao R. (2020) ShinyGO: a graphical gene-set enrichment tool for animals and plants. Bioinformatics **36**, 2628–2629

Gerault MA, Camoin L, Granjeaud S. (2024) DIAgui: a Shiny application to process the output from DIA-NN. Bioinform Adv. **4**, vbae001.

Han L, Usher S, Sandgrind S, Hassall K, Sayanova O, Michaelson LV, Haslam RP, Napier JA. (2020) High level accumulation of EPA and DHA in field-grown transgenic Camelina - a multi-territory evaluation of TAG accumulation and heterogeneity. Plant Biotechnol J. **18**, 2280-2291.

Han L, Silvestre S, Sayanova O, Haslam RP, Napier JA. (2022) Using field evaluation and systematic iteration to rationalize the accumulation of omega-3 long-chain polyunsaturated fatty acids in transgenic *Camelina sativa*. Plant Biotechnol J. **20**,1833-1852.

Nguyen HT, Silva JE, Podicheti R, Macrander J, Yang W, Nazarenus TJ, Nam JW, Jaworski JG, Lu C, Scheffler BE, Mockaitis K, Cahoon EB. (2013) Camelina seed transcriptome: a tool for meal and oil improvement and translational research. Plant Biotechnol J. 11(6):759-69.

Perez-Riverol Y, Bai J, Bandla C, García-Seisdedos D, Hewapathirana S, Kamatchinathan S, Kundu DJ, Prakash A, Frericks-Zipper A, Eisenacher M, et al (2022) The PRIDE database resources in 2022: a hub for mass spectrometry-based proteomics evidences. Nucleic Acids Res **50**, D543–D552

Pollard M, Martin TM, Shachar-Hill Y. (2015) Lipid analysis of developing Camelina sativa seeds and cultured embryos. Phytochemistry. 118, 23-32.

Russo, R., Reggiani, R. (2015) Seed protein in Camelina sativa (L.) Cranz var. Calena. Int. J. Plant Soil Sci. 8, 1-6

Zhang, H., Gannon, L., Hassall, K.L., Deery, M., Gibbs, D.J., Holdsworth, M.J., van der Hoorn, R.A.L., Lilley, K.S, Theodoulou, F.L. (2018) N-terminomics reveals control of Arabidopsis storage reserves and proteases by the Arg/N-end rule pathway. New Phytol., **218**, 1106-1126

Zhong, Z., Bailey, M., Kim, YI. et al. (2025) The distinct roles of genome, methylation, transcription, and translation on protein expression in *Arabidopsis thaliana* resolve the Central Dogma’s information flow. Genome Biol. **26**, 319
