# Supplementary material for: Assessing the Impact of Multigene Engineering on the Proteome: Omega‐3 Camelina as a Case Study
Source: Plant Biotechnol J. 2026 Jul 13:10.1111/pbi.70712. Online ahead of print. doi: 10.1111/pbi.70712 (PMC13399257; doi:10.1111/pbi.70712)
Supplement: Supplementary file 4 — Figure S1: Details of transgenic lines. Figure S2: Tandem mass tag (TMT) label‐based quantitative proteomics workflow. Figure S3: Transgene encoded proteins identified using TMT label‐based proteomics. Figure S4: Differential abundance of lipid metabolic enzymes in Camelina seed engineered to produce EPA and DHA. Figure S5: Differential abundance of ubiquitin proteasome‐related proteins in Camelina seed engineered to produce EPA and DHA. Figure S6: DIA label‐free quantitative proteomics workflow. Figure S7: Camelina seed proteins identified and quantified using MaxLFQ label‐free quantification. Figure S8: Transgene encoded proteins identified using label‐free proteomics. Figure S9: Normalised intensity‐based absolute quantification (iBAQ) of Camelina seed proteins. [file PBI-9999-0-s004.pptx]

## Slide 1
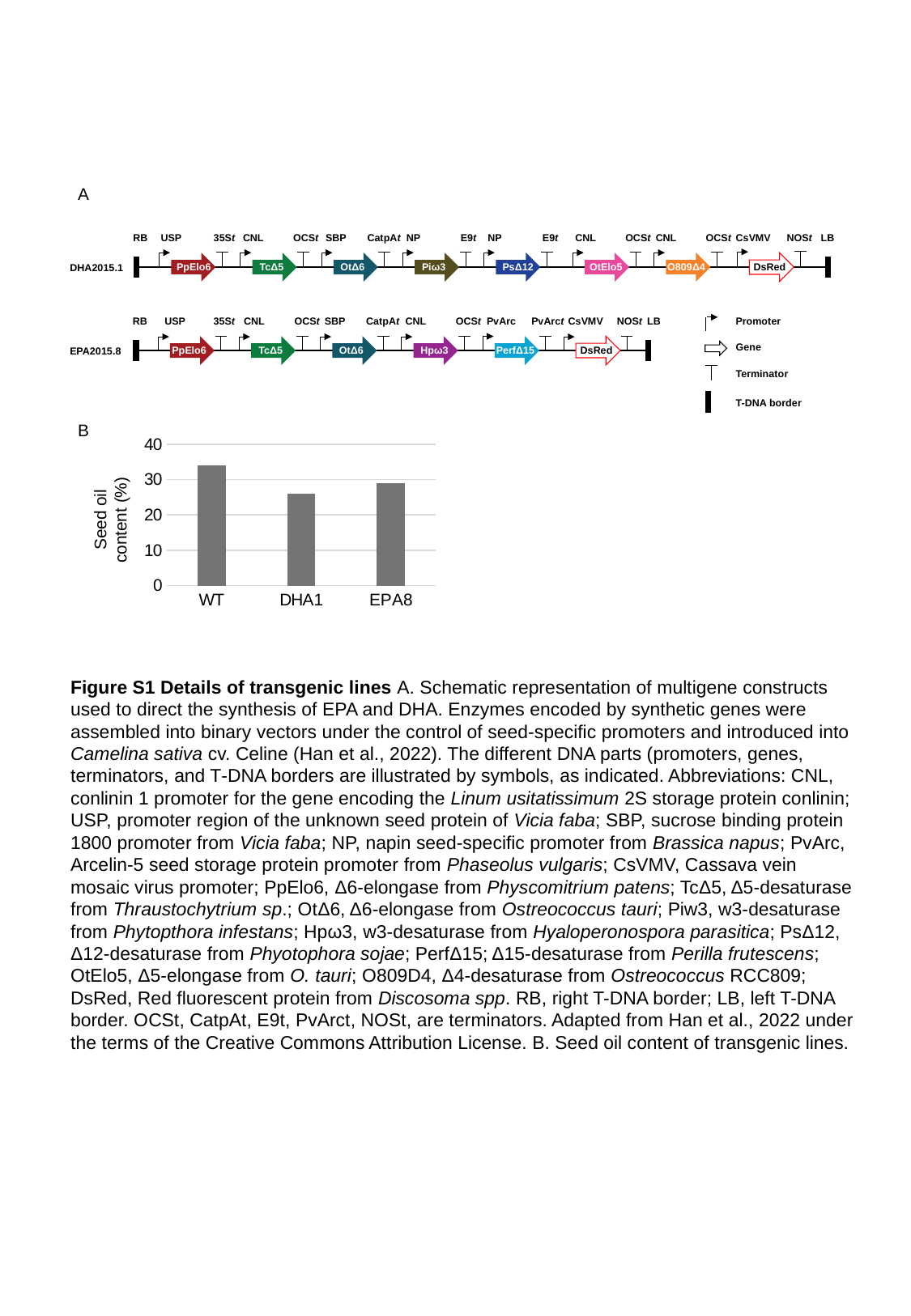

A
RB
USP
35St
CNL
OCSt
SBP
CatpAt
NP
E9t
NP
E9t
CNL
OCSt
CNL
OCSt
CsVMV
NOSt
LB
PpElo6
TcΔ5
OtΔ6
Piω3
PsΔ12
OtElo5
O809Δ4
DsRed
DHA2015.1
Promoter
RB
35St
OCSt
SBP
CatpAt
CNL
OCSt
PvArc
PvArct
CsVMV
NOSt
LB
USP
CNL
Gene
PpElo6
TcΔ5
OtΔ6
Hpω3
PerfΔ15
DsRed
EPA2015.8
Terminator
T-DNA border
B
### Chart
| Category | SoC (%) |
|---|---|
| WT | 34.0 |
| DHA1 | 26.0 |
| EPA8 | 29.0 |Seed oil content (%)
Figure S1 Details of transgenic lines A. Schematic representation of multigene constructs used to direct the synthesis of EPA and DHA. Enzymes encoded by synthetic genes were assembled into binary vectors under the control of seed‐specific promoters and introduced into Camelina sativa cv. Celine (Han et al., 2022). The different DNA parts (promoters, genes, terminators, and T‐DNA borders are illustrated by symbols, as indicated. Abbreviations: CNL, conlinin 1 promoter for the gene encoding the Linum usitatissimum 2S storage protein conlinin; USP, promoter region of the unknown seed protein of Vicia faba; SBP, sucrose binding protein 1800 promoter from Vicia faba; NP, napin seed‐specific promoter from Brassica napus; PvArc, Arcelin‐5 seed storage protein promoter from Phaseolus vulgaris; CsVMV, Cassava vein mosaic virus promoter; PpElo6, Δ6‐elongase from Physcomitrium patens; TcΔ5, Δ5‐desaturase from Thraustochytrium sp.; OtΔ6, Δ6‐elongase from Ostreococcus tauri; Piw3, w3‐desaturase from Phytopthora infestans; Hpω3, w3‐desaturase from Hyaloperonospora parasitica; PsΔ12, Δ12‐desaturase from Phyotophora sojae; PerfΔ15; Δ15‐desaturase from Perilla frutescens; OtElo5, Δ5‐elongase from O. tauri; O809D4, Δ4‐desaturase from Ostreococcus RCC809; DsRed, Red fluorescent protein from Discosoma spp. RB, right T-DNA border; LB, left T-DNA border. OCSt, CatpAt, E9t, PvArct, NOSt, are terminators. Adapted from Han et al., 2022 under the terms of the Creative Commons Attribution License. B. Seed oil content of transgenic lines.

## Slide 2
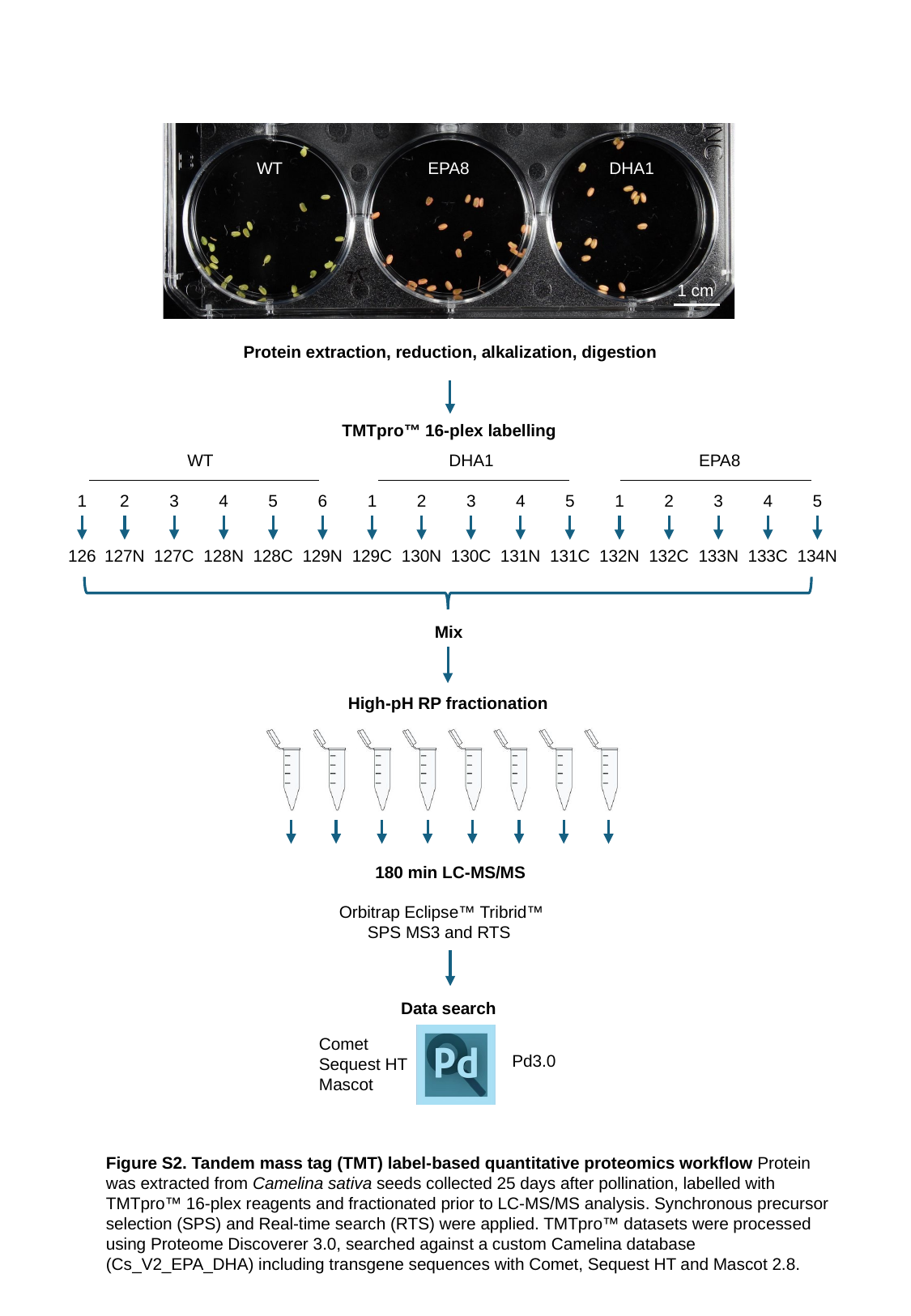

WT
EPA8
DHA1
1 cm
Protein extraction, reduction, alkalization, digestion
TMTpro™ 16-plex labelling
WT
DHA1
EPA8
1
2
3
4
5
6
1
2
3
4
5
1
2
3
4
5
126
127N
127C
128N
128C
129N
129C
130N
130C
131N
131C
132N
132C
133N
133C
134N
Mix
High-pH RP fractionation
180 min LC-MS/MS
Orbitrap Eclipse™ Tribrid™
SPS MS3 and RTS
Data search
Comet
Sequest HT
Mascot
Pd3.0
Figure S2. Tandem mass tag (TMT) label-based quantitative proteomics workflow Protein was extracted from Camelina sativa seeds collected 25 days after pollination, labelled with TMTpro™ 16-plex reagents and fractionated prior to LC-MS/MS analysis. Synchronous precursor selection (SPS) and Real‑time search (RTS) were applied. TMTpro™ datasets were processed using Proteome Discoverer 3.0, searched against a custom Camelina database (Cs_V2_EPA_DHA) including transgene sequences with Comet, Sequest HT and Mascot 2.8.

## Slide 3
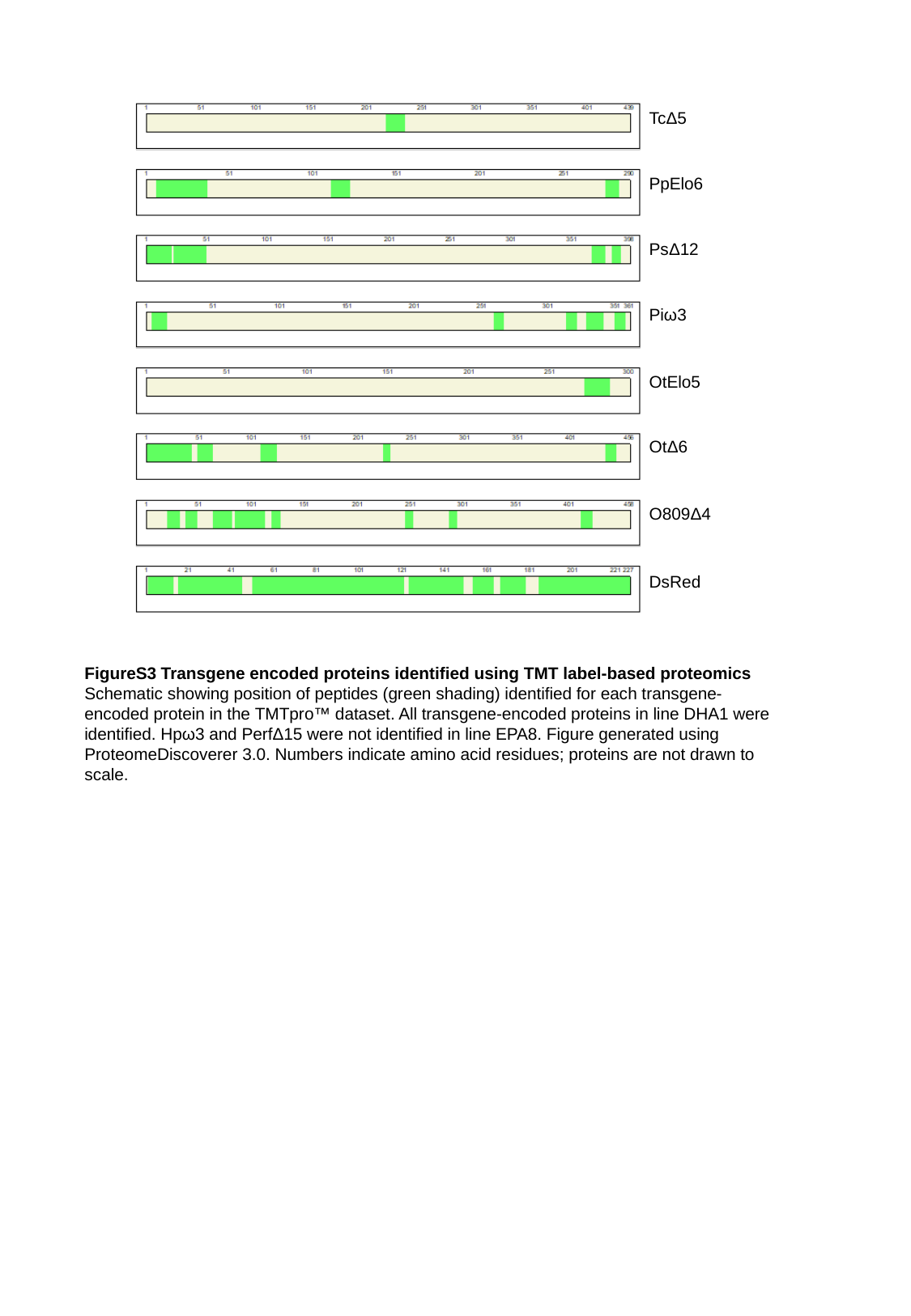

TcΔ5
PpElo6
PsΔ12
Piω3
OtElo5
OtΔ6
O809Δ4
DsRed
FigureS3 Transgene encoded proteins identified using TMT label-based proteomics
Schematic showing position of peptides (green shading) identified for each transgene-encoded protein in the TMTpro™ dataset. All transgene-encoded proteins in line DHA1 were identified. Hpω3 and PerfΔ15 were not identified in line EPA8. Figure generated using ProteomeDiscoverer 3.0. Numbers indicate amino acid residues; proteins are not drawn to scale.

## Slide 4
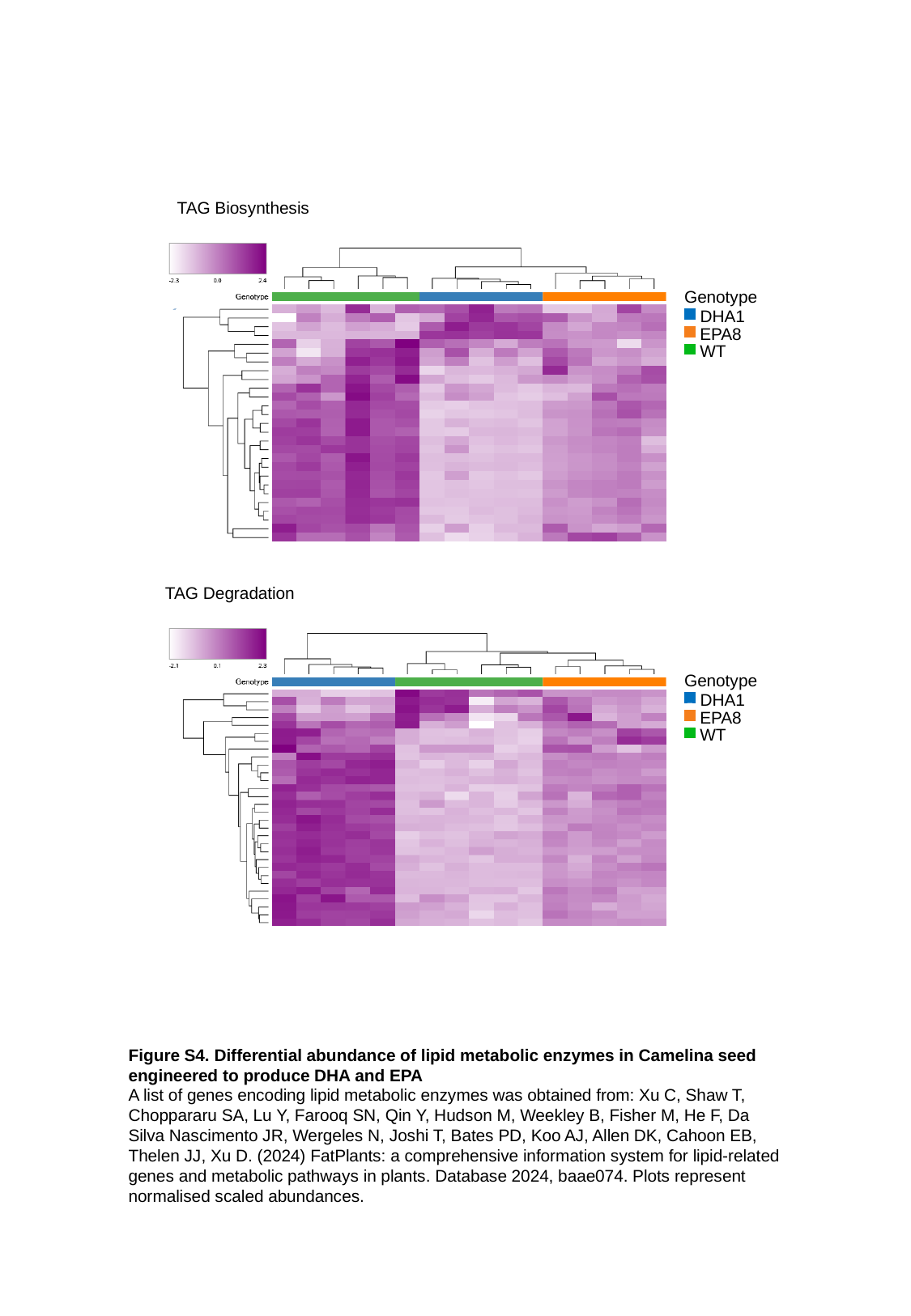

TAG Biosynthesis
Genotype
DHA1
EPA8
WT
TAG Degradation
Genotype
DHA1
EPA8
WT
Figure S4. Differential abundance of lipid metabolic enzymes in Camelina seed engineered to produce DHA and EPA
A list of genes encoding lipid metabolic enzymes was obtained from: Xu C, Shaw T, Choppararu SA, Lu Y, Farooq SN, Qin Y, Hudson M, Weekley B, Fisher M, He F, Da Silva Nascimento JR, Wergeles N, Joshi T, Bates PD, Koo AJ, Allen DK, Cahoon EB, Thelen JJ, Xu D. (2024) FatPlants: a comprehensive information system for lipid-related genes and metabolic pathways in plants. Database 2024, baae074. Plots represent normalised scaled abundances.

## Slide 5
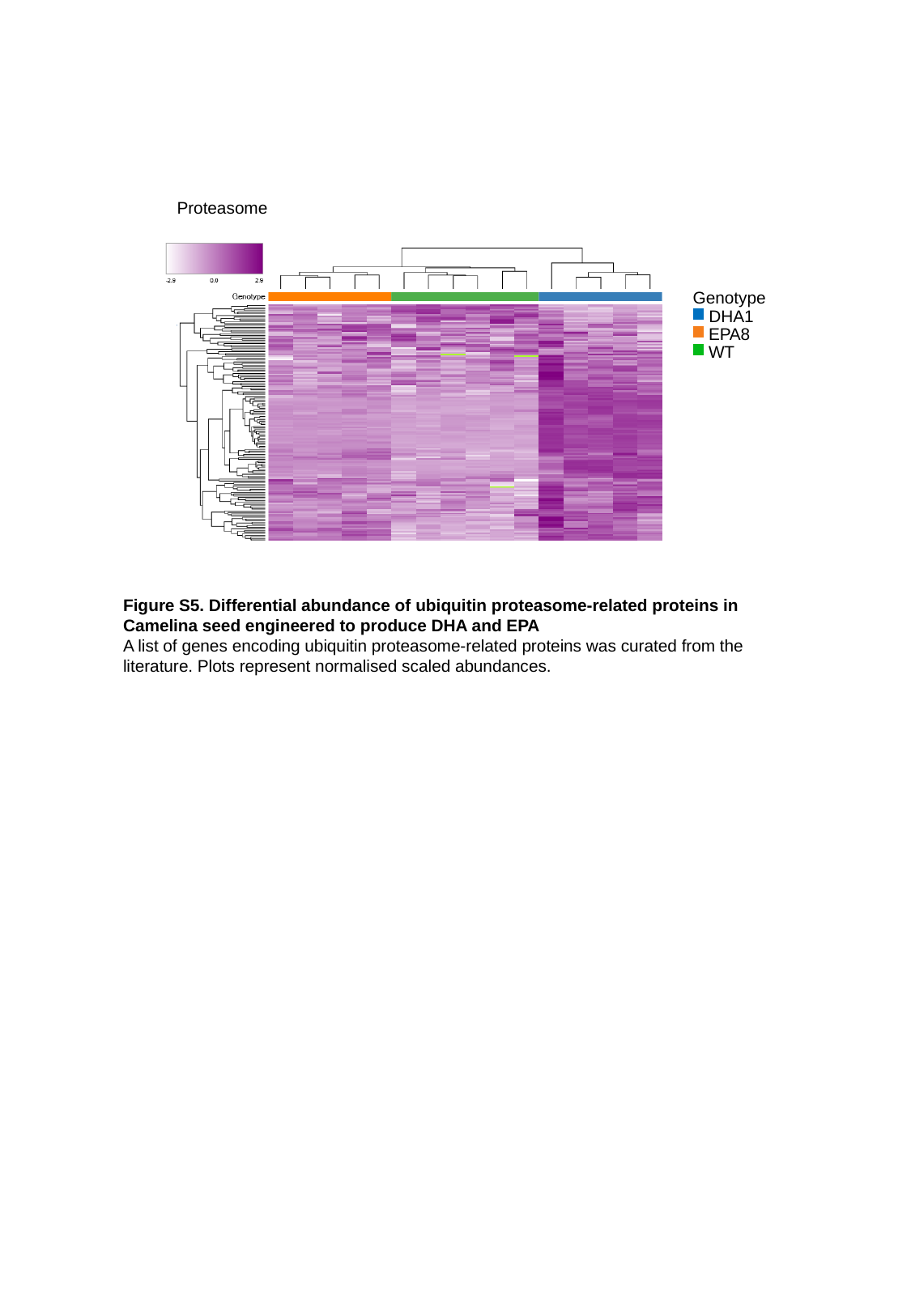

Proteasome
Genotype
DHA1
EPA8
WT
Figure S5. Differential abundance of ubiquitin proteasome-related proteins in Camelina seed engineered to produce DHA and EPA
A list of genes encoding ubiquitin proteasome-related proteins was curated from the literature. Plots represent normalised scaled abundances.

## Slide 6
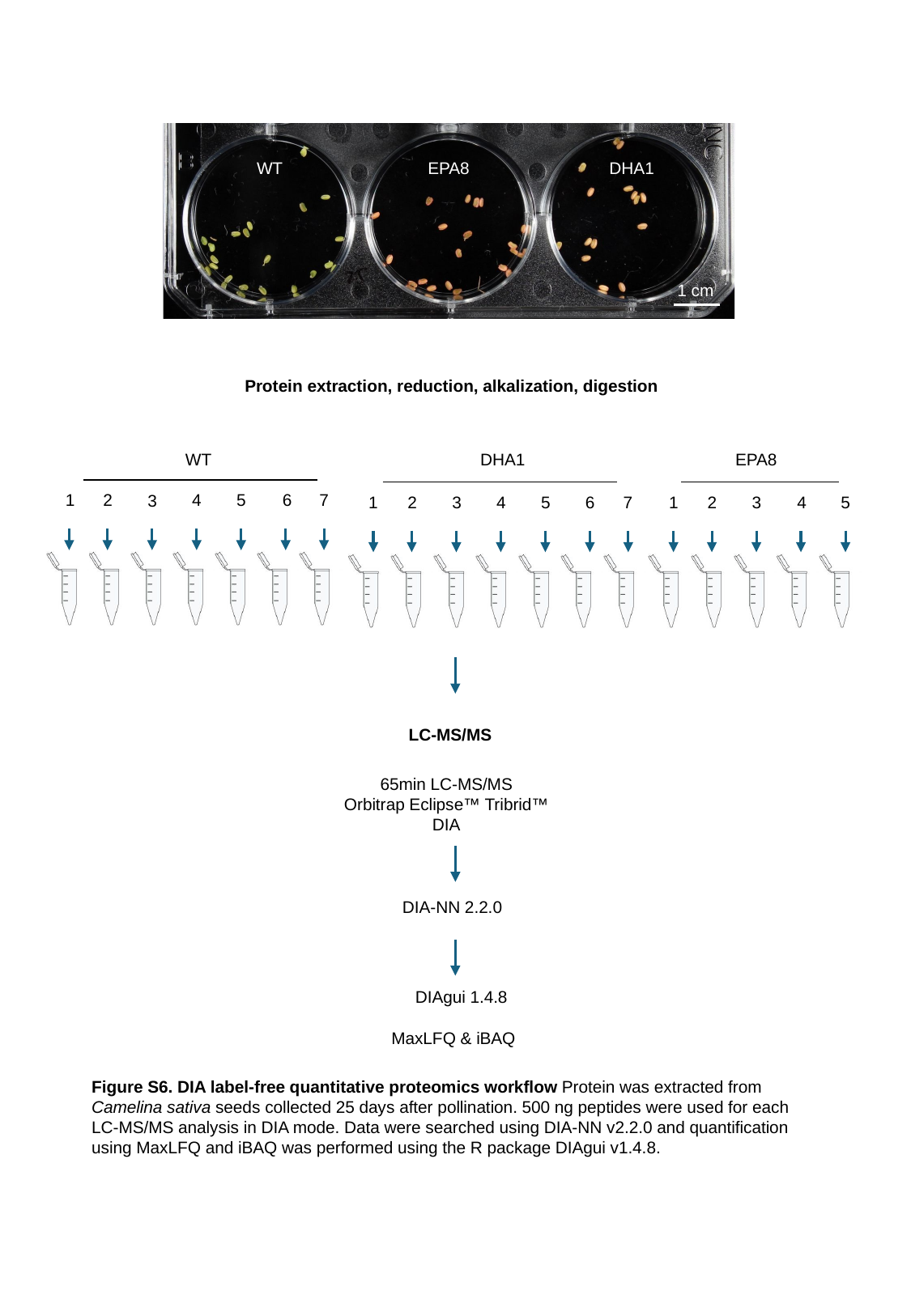

WT
EPA8
DHA1
1 cm
Protein extraction, reduction, alkalization, digestion
WT
DHA1
EPA8
1
2
4
5
6
7
3
1
2
3
4
5
6
7
1
2
3
4
5
LC-MS/MS
65min LC-MS/MS
Orbitrap Eclipse™ Tribrid™
DIA
DIA-NN 2.2.0
DIAgui 1.4.8
MaxLFQ & iBAQ
Figure S6. DIA label-free quantitative proteomics workflow Protein was extracted from Camelina sativa seeds collected 25 days after pollination. 500 ng peptides were used for each LC-MS/MS analysis in DIA mode. Data were searched using DIA-NN v2.2.0 and quantification using MaxLFQ and iBAQ was performed using the R package DIAgui v1.4.8.

## Slide 7
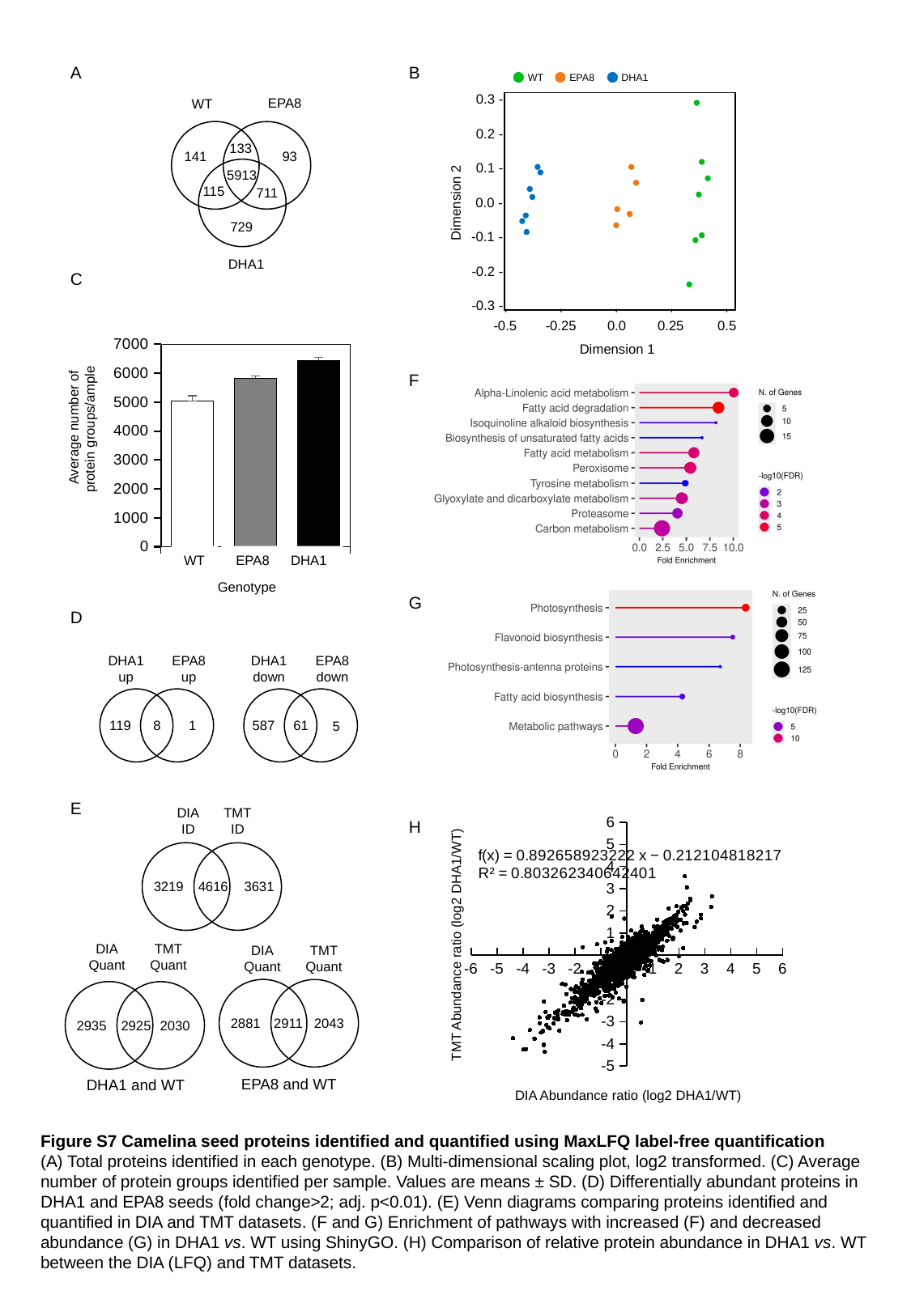

A
B
WT
EPA8
DHA1
0.3 -
0.2 -
0.1 -
0.0 -
Dimension 2
-0.1 -
-0.2 -
-0.3 -
-
-
-
-
-
-0.5
-0.25
0.0
0.25
0.5
Dimension 1
EPA8
WT
133
141
93
5913
115
711
729
DHA1
C
### Chart
| Category | |
|---|---|
| WT | 5031.857142857143 |
| EPA | 5827.8 |
| DHA | 6440.857142857143 |Average number of protein groups/ample
Genotype
F
WT
EPA8
DHA1
G
D
DHA1 up
EPA8 up
DHA1 down
EPA8 down
119
8
1
587
61
5
E
DIA
ID
TMT
ID
### Chart
| Category | Abundance Ratio (log2): (DHA) / (WT) |
|---|---|H
3219
4616
3631
TMT Abundance ratio (log2 DHA1/WT)
DIA
Quant
TMT
Quant
DIA
Quant
TMT
Quant
2881
2911
2043
2935
2925
2030
EPA8 and WT
DHA1 and WT
DIA Abundance ratio (log2 DHA1/WT)
Figure S7 Camelina seed proteins identified and quantified using MaxLFQ label-free quantification
(A) Total proteins identified in each genotype. (B) Multi-dimensional scaling plot, log2 transformed. (C) Average number of protein groups identified per sample. Values are means ± SD. (D) Differentially abundant proteins in DHA1 and EPA8 seeds (fold change>2; adj. p<0.01). (E) Venn diagrams comparing proteins identified and quantified in DIA and TMT datasets. (F and G) Enrichment of pathways with increased (F) and decreased abundance (G) in DHA1 vs. WT using ShinyGO. (H) Comparison of relative protein abundance in DHA1 vs. WT between the DIA (LFQ) and TMT datasets.

## Slide 8
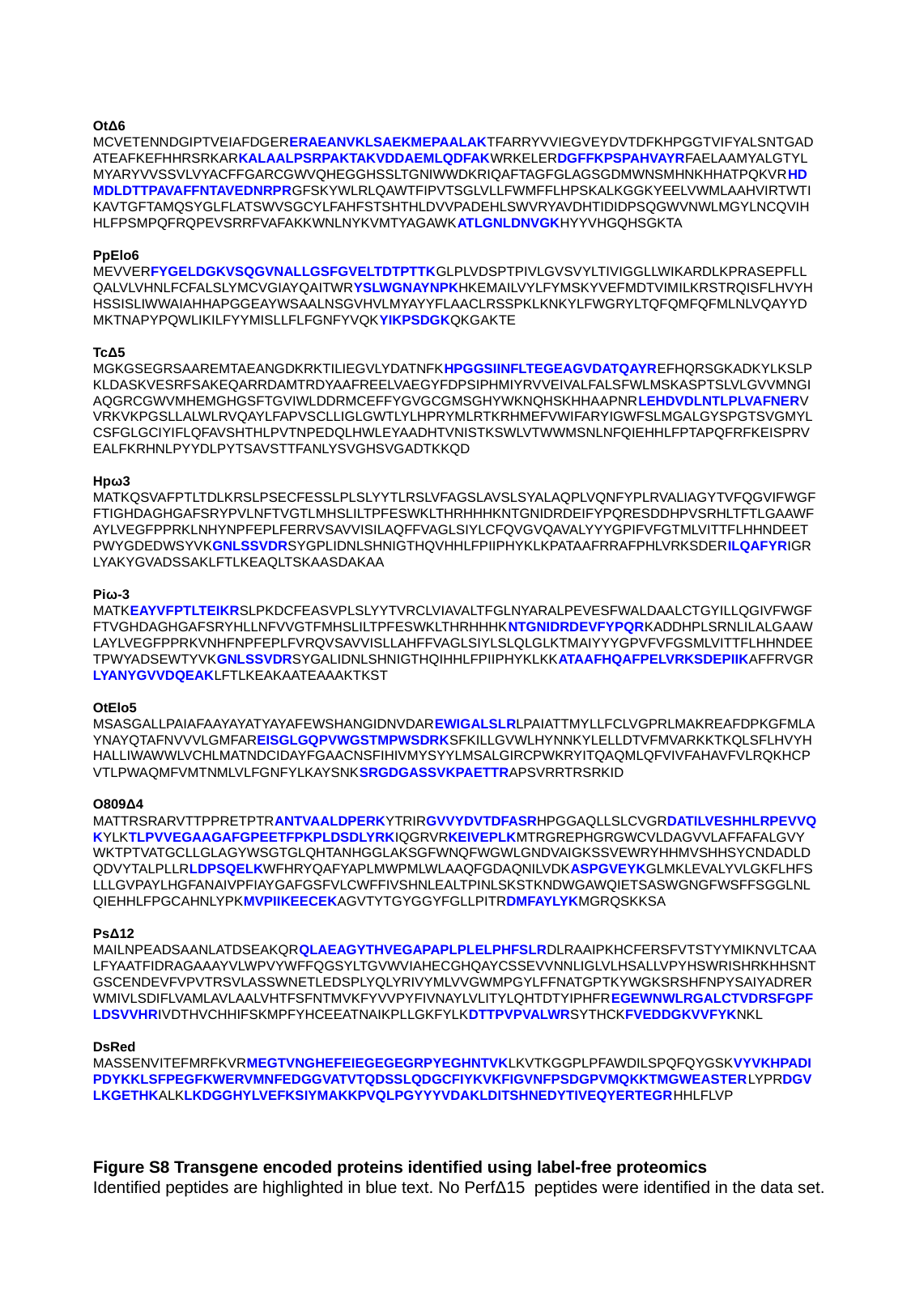

OtΔ6
MCVETENNDGIPTVEIAFDGERERAEANVKLSAEKMEPAALAKTFARRYVVIEGVEYDVTDFKHPGGTVIFYALSNTGADATEAFKEFHHRSRKARKALAALPSRPAKTAKVDDAEMLQDFAKWRKELERDGFFKPSPAHVAYRFAELAAMYALGTYLMYARYVVSSVLVYACFFGARCGWVQHEGGHSSLTGNIWWDKRIQAFTAGFGLAGSGDMWNSMHNKHHATPQKVRHDMDLDTTPAVAFFNTAVEDNRPRGFSKYWLRLQAWTFIPVTSGLVLLFWMFFLHPSKALKGGKYEELVWMLAAHVIRTWTIKAVTGFTAMQSYGLFLATSWVSGCYLFAHFSTSHTHLDVVPADEHLSWVRYAVDHTIDIDPSQGWVNWLMGYLNCQVIHHLFPSMPQFRQPEVSRRFVAFAKKWNLNYKVMTYAGAWKATLGNLDNVGKHYYVHGQHSGKTA
PpElo6
MEVVERFYGELDGKVSQGVNALLGSFGVELTDTPTTKGLPLVDSPTPIVLGVSVYLTIVIGGLLWIKARDLKPRASEPFLLQALVLVHNLFCFALSLYMCVGIAYQAITWRYSLWGNAYNPKHKEMAILVYLFYMSKYVEFMDTVIMILKRSTRQISFLHVYHHSSISLIWWAIAHHAPGGEAYWSAALNSGVHVLMYAYYFLAACLRSSPKLKNKYLFWGRYLTQFQMFQFMLNLVQAYYDMKTNAPYPQWLIKILFYYMISLLFLFGNFYVQKYIKPSDGKQKGAKTE
TcΔ5
MGKGSEGRSAAREMTAEANGDKRKTILIEGVLYDATNFKHPGGSIINFLTEGEAGVDATQAYREFHQRSGKADKYLKSLPKLDASKVESRFSAKEQARRDAMTRDYAAFREELVAEGYFDPSIPHMIYRVVEIVALFALSFWLMSKASPTSLVLGVVMNGIAQGRCGWVMHEMGHGSFTGVIWLDDRMCEFFYGVGCGMSGHYWKNQHSKHHAAPNRLEHDVDLNTLPLVAFNERVVRKVKPGSLLALWLRVQAYLFAPVSCLLIGLGWTLYLHPRYMLRTKRHMEFVWIFARYIGWFSLMGALGYSPGTSVGMYLCSFGLGCIYIFLQFAVSHTHLPVTNPEDQLHWLEYAADHTVNISTKSWLVTWWMSNLNFQIEHHLFPTAPQFRFKEISPRVEALFKRHNLPYYDLPYTSAVSTTFANLYSVGHSVGADTKKQD
Hpω3
MATKQSVAFPTLTDLKRSLPSECFESSLPLSLYYTLRSLVFAGSLAVSLSYALAQPLVQNFYPLRVALIAGYTVFQGVIFWGFFTIGHDAGHGAFSRYPVLNFTVGTLMHSLILTPFESWKLTHRHHHKNTGNIDRDEIFYPQRESDDHPVSRHLTFTLGAAWFAYLVEGFPPRKLNHYNPFEPLFERRVSAVVISILAQFFVAGLSIYLCFQVGVQAVALYYYGPIFVFGTMLVITTFLHHNDEETPWYGDEDWSYVKGNLSSVDRSYGPLIDNLSHNIGTHQVHHLFPIIPHYKLKPATAAFRRAFPHLVRKSDERILQAFYRIGRLYAKYGVADSSAKLFTLKEAQLTSKAASDAKAA
Piω-3
MATKEAYVFPTLTEIKRSLPKDCFEASVPLSLYYTVRCLVIAVALTFGLNYARALPEVESFWALDAALCTGYILLQGIVFWGFFTVGHDAGHGAFSRYHLLNFVVGTFMHSLILTPFESWKLTHRHHHKNTGNIDRDEVFYPQRKADDHPLSRNLILALGAAWLAYLVEGFPPRKVNHFNPFEPLFVRQVSAVVISLLAHFFVAGLSIYLSLQLGLKTMAIYYYGPVFVFGSMLVITTFLHHNDEETPWYADSEWTYVKGNLSSVDRSYGALIDNLSHNIGTHQIHHLFPIIPHYKLKKATAAFHQAFPELVRKSDEPIIKAFFRVGRLYANYGVVDQEAKLFTLKEAKAATEAAAKTKST
OtElo5
MSASGALLPAIAFAAYAYATYAYAFEWSHANGIDNVDAREWIGALSLRLPAIATTMYLLFCLVGPRLMAKREAFDPKGFMLAYNAYQTAFNVVVLGMFAREISGLGQPVWGSTMPWSDRKSFKILLGVWLHYNNKYLELLDTVFMVARKKTKQLSFLHVYHHALLIWAWWLVCHLMATNDCIDAYFGAACNSFIHIVMYSYYLMSALGIRCPWKRYITQAQMLQFVIVFAHAVFVLRQKHCPVTLPWAQMFVMTNMLVLFGNFYLKAYSNKSRGDGASSVKPAETTRAPSVRRTRSRKID
O809Δ4
MATTRSRARVTTPPRETPTRANTVAALDPERKYTRIRGVVYDVTDFASRHPGGAQLLSLCVGRDATILVESHHLRPEVVQKYLKTLPVVEGAAGAFGPEETFPKPLDSDLYRKIQGRVRKEIVEPLKMTRGREPHGRGWCVLDAGVVLAFFAFALGVYWKTPTVATGCLLGLAGYWSGTGLQHTANHGGLAKSGFWNQFWGWLGNDVAIGKSSVEWRYHHMVSHHSYCNDADLDQDVYTALPLLRLDPSQELKWFHRYQAFYAPLMWPMLWLAAQFGDAQNILVDKASPGVEYKGLMKLEVALYVLGKFLHFSLLLGVPAYLHGFANAIVPFIAYGAFGSFVLCWFFIVSHNLEALTPINLSKSTKNDWGAWQIETSASWGNGFWSFFSGGLNLQIEHHLFPGCAHNLYPKMVPIIKEECEKAGVTYTGYGGYFGLLPITRDMFAYLYKMGRQSKKSA
PsΔ12
MAILNPEADSAANLATDSEAKQRQLAEAGYTHVEGAPAPLPLELPHFSLRDLRAAIPKHCFERSFVTSTYYMIKNVLTCAALFYAATFIDRAGAAAYVLWPVYWFFQGSYLTGVWVIAHECGHQAYCSSEVVNNLIGLVLHSALLVPYHSWRISHRKHHSNTGSCENDEVFVPVTRSVLASSWNETLEDSPLYQLYRIVYMLVVGWMPGYLFFNATGPTKYWGKSRSHFNPYSAIYADRERWMIVLSDIFLVAMLAVLAALVHTFSFNTMVKFYVVPYFIVNAYLVLITYLQHTDTYIPHFREGEWNWLRGALCTVDRSFGPFLDSVVHRIVDTHVCHHIFSKMPFYHCEEATNAIKPLLGKFYLKDTTPVPVALWRSYTHCKFVEDDGKVVFYKNKL
DsRed
MASSENVITEFMRFKVRMEGTVNGHEFEIEGEGEGRPYEGHNTVKLKVTKGGPLPFAWDILSPQFQYGSKVYVKHPADIPDYKKLSFPEGFKWERVMNFEDGGVATVTQDSSLQDGCFIYKVKFIGVNFPSDGPVMQKKTMGWEASTERLYPRDGVLKGETHKALKLKDGGHYLVEFKSIYMAKKPVQLPGYYYVDAKLDITSHNEDYTIVEQYERTEGRHHLFLVP
Figure S8 Transgene encoded proteins identified using label-free proteomics
Identified peptides are highlighted in blue text. No PerfΔ15 peptides were identified in the data set.

## Slide 9
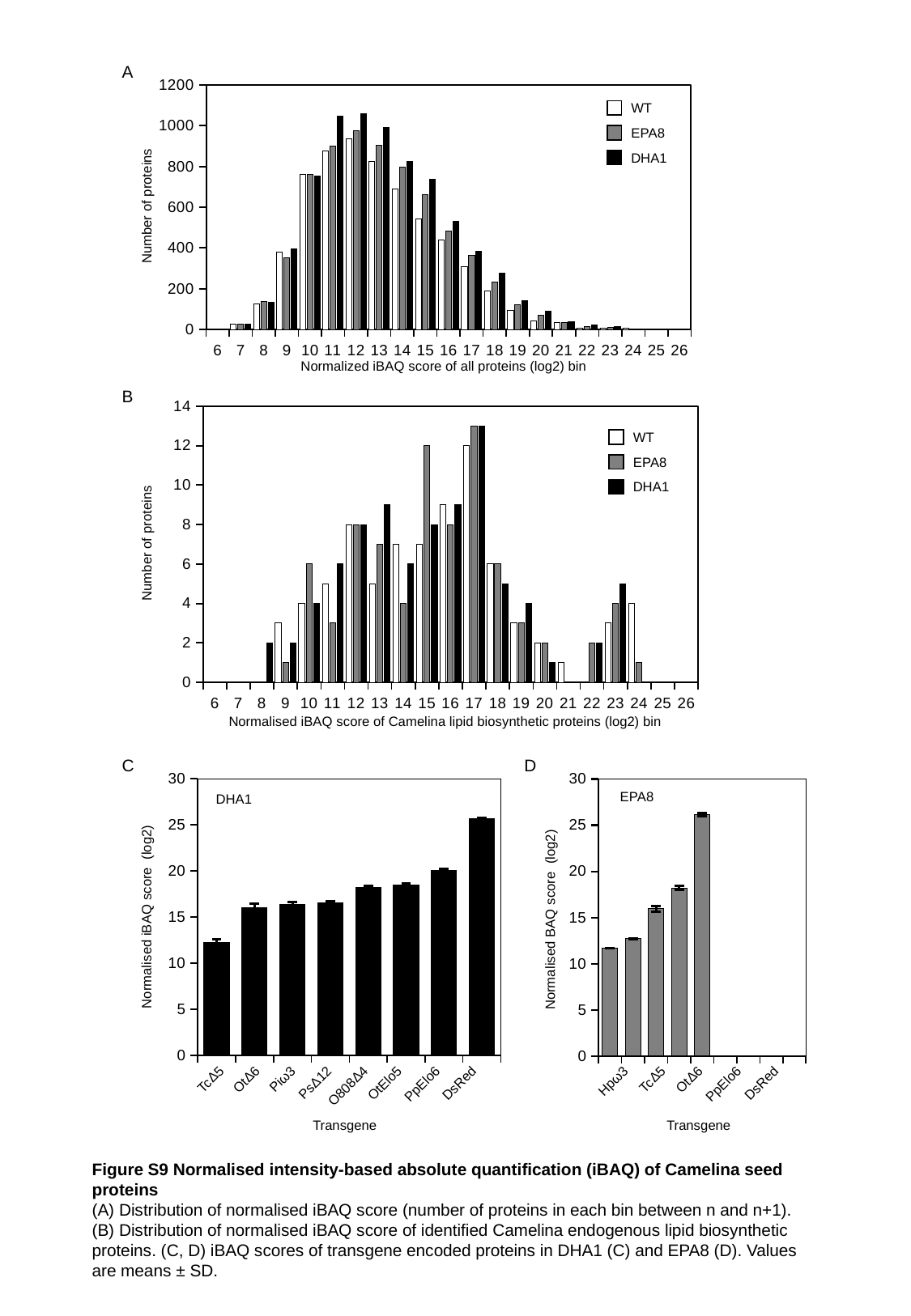

A
### Chart
| Category | WT | EPA | DHA |
|---|---|---|---|
| 6 | 2.0 | 2.0 | 2.0 |
| 7 | 26.0 | 27.0 | 28.0 |
| 8 | 126.0 | 137.0 | 132.0 |
| 9 | 381.0 | 351.0 | 395.0 |
| 10 | 762.0 | 761.0 | 752.0 |
| 11 | 876.0 | 899.0 | 1049.0 |
| 12 | 937.0 | 975.0 | 1058.0 |
| 13 | 823.0 | 903.0 | 993.0 |
| 14 | 690.0 | 798.0 | 825.0 |
| 15 | 544.0 | 662.0 | 736.0 |
| 16 | 441.0 | 482.0 | 532.0 |
| 17 | 308.0 | 365.0 | 382.0 |
| 18 | 190.0 | 232.0 | 276.0 |
| 19 | 93.0 | 124.0 | 142.0 |
| 20 | 42.0 | 69.0 | 90.0 |
| 21 | 36.0 | 35.0 | 39.0 |
| 22 | 8.0 | 14.0 | 21.0 |
| 23 | 8.0 | 9.0 | 13.0 |
| 24 | 6.0 | 1.0 | 2.0 |
| 25 | 1.0 | 3.0 | 1.0 |
| 26 | 2.0 | 1.0 | 0.0 |WT
EPA8
DHA1
Number of proteins
Normalized iBAQ score of all proteins (log2) bin
B
### Chart
| Category | WT | EPA | DHA |
|---|---|---|---|
| 6 | 0.0 | 0.0 | 0.0 |
| 7 | 0.0 | 0.0 | 0.0 |
| 8 | 0.0 | 0.0 | 2.0 |
| 9 | 3.0 | 1.0 | 2.0 |
| 10 | 4.0 | 6.0 | 4.0 |
| 11 | 5.0 | 3.0 | 6.0 |
| 12 | 8.0 | 8.0 | 8.0 |
| 13 | 5.0 | 7.0 | 9.0 |
| 14 | 7.0 | 4.0 | 6.0 |
| 15 | 7.0 | 12.0 | 8.0 |
| 16 | 9.0 | 8.0 | 9.0 |
| 17 | 12.0 | 13.0 | 13.0 |
| 18 | 6.0 | 6.0 | 5.0 |
| 19 | 3.0 | 3.0 | 4.0 |
| 20 | 2.0 | 2.0 | 1.0 |
| 21 | 1.0 | 0.0 | 0.0 |
| 22 | 0.0 | 2.0 | 2.0 |
| 23 | 3.0 | 4.0 | 5.0 |
| 24 | 4.0 | 1.0 | 0.0 |
| 25 | 0.0 | 0.0 | 0.0 |
| 26 | 0.0 | 0.0 | 0.0 |WT
EPA8
DHA1
Number of proteins
Normalised iBAQ score of Camelina lipid biosynthetic proteins (log2) bin
C
D
### Chart
| Category | |
|---|---|
| Tc?5 | 12.225874972233852 |
| Ot?6 | 16.00978046014566 |
| Piw-3 | 16.32930645136401 |
| Ps?12 | 16.499945682663405 |
| O809?4 | 18.186560654087575 |
| OtElo5 | 18.456399123386028 |
| PSE1 | 20.03489683715383 |
| DsRed | 25.624681941553487 |
[unsupported chart]
EPA8
DHA1
 Normalised iBAQ score (log2)
Normalised BAQ score (log2)
Piω3
OtΔ6
OtΔ6
TcΔ5
TcΔ5
Hpω3
DsRed
OtElo5
DsRed
PsΔ12
PpElo6
PpElo6
O808Δ4
Transgene
Transgene
Figure S9 Normalised intensity-based absolute quantification (iBAQ) of Camelina seed proteins
(A) Distribution of normalised iBAQ score (number of proteins in each bin between n and n+1). (B) Distribution of normalised iBAQ score of identified Camelina endogenous lipid biosynthetic proteins. (C, D) iBAQ scores of transgene encoded proteins in DHA1 (C) and EPA8 (D). Values are means ± SD.
